# Perspectives on social health among patients from Arab backgrounds receiving kidney replacement therapy: an interview study

**DOI:** 10.1093/ckj/sfaf081

**Published:** 2025-03-13

**Authors:** Nibras Jasim, Amanda Sluiter, Mary Ann Nicdao, Chandana Guha, Allison Jaure, Nicole Scholes-Robertson, Ben J Smith, Germaine Wong, Karine Manera

**Affiliations:** Research and Education Network, Western Sydney Local Health District, Sydney, Australia; Centre for Kidney Research, The Children's Hospital at Westmead, Sydney, Australia; Centre for Kidney Research, The Children's Hospital at Westmead, Sydney, Australia; Sydney School of Public Health, The University of Sydney, Sydney, Australia; Centre for Kidney Research, The Children's Hospital at Westmead, Sydney, Australia; Sydney School of Public Health, The University of Sydney, Sydney, Australia; Centre for Kidney Research, The Children's Hospital at Westmead, Sydney, Australia; Sydney School of Public Health, The University of Sydney, Sydney, Australia; Centre for Kidney Research, The Children's Hospital at Westmead, Sydney, Australia; Sydney School of Public Health, The University of Sydney, Sydney, Australia; Centre for Kidney Research, The Children's Hospital at Westmead, Sydney, Australia; Sydney School of Public Health, The University of Sydney, Sydney, Australia; Research and Education Network, Western Sydney Local Health District, Sydney, Australia; Sydney School of Public Health, The University of Sydney, Sydney, Australia; Centre for Kidney Research, The Children's Hospital at Westmead, Sydney, Australia; Sydney School of Public Health, The University of Sydney, Sydney, Australia; Department of Renal Medicine, Westmead Hospital, Western Sydney Local Health District, Sydney, Australia; Research and Education Network, Western Sydney Local Health District, Sydney, Australia; Centre for Kidney Research, The Children's Hospital at Westmead, Sydney, Australia; Sydney School of Public Health, The University of Sydney, Sydney, Australia

**Keywords:** chronic kidney disease, kidney replacement therapy, loneliness, social health, social isolation

## Abstract

**Background:**

Patients with chronic kidney disease receiving kidney replacement therapy have an increased risk of having poor social participation and connections, which are associated with poor health outcomes. This may be exacerbated in people from minority or disadvantaged groups, including culturally and linguistically diverse populations, who face multiple social disadvantages. We aimed to describe the perspectives on social health and connections among patients from Arab backgrounds receiving kidney replacement therapy.

**Methods:**

Semi-structured interviews were conducted in Arabic or English language with Arab Australians receiving kidney replacement therapy across four renal units within the Western Renal Service, Australia. Transcripts were thematically analysed.

**Results:**

Twenty-five participants were interviewed, including 13 (52%) male and 22 (88%) born overseas. Four themes were identified: treatment impeding social participation (missing out on family time, limited opportunity for close friendships, symptoms interfering with relationships, reluctant to socialize to avoid infection); diminishing community and cultural ties (stigmatized and ostracized, geographic and cultural separation, emotional disconnect, avoiding additional distress); struggling with loss of normality within the family (inability to fulfil gender roles, hiding pain to protect children); and deriving comfort from connection (cultural norms preventing loneliness, easing the burden with support from family and friends, kinship and companionship during in-centre dialysis, using technology to connect with others).

**Conclusions:**

Patients from Arab backgrounds face substantial barriers to social participation, leading to loss of connection with people and culture. Strategies to improve social connections through culturally tailored peer and family support are needed.

KEY LEARNING POINTS **What was known:**

- Patients receiving kidney replacement therapy are restricted in their social participation due to the disease and treatment burden.
- Minority groups, especially those from culturally and linguistically diverse backgrounds, face higher risk of chronic kidney disease and social isolation, compounding their risk of poor health outcomes.
- Patients from culturally and linguistically diverse backgrounds are under-represented in research, and evidence of the experiences and social impacts of kidney replacement therapy is lacking.

**This study adds:**

- Patients receiving kidney replacement therapy from Arab communities face barriers to social participation, including time lost to dialysis and reduced engagement with family, friends, and community.
- Disrupted social and gender roles led to distress within families, while stigma, travel limitations, and symptoms like fatigue impacted cultural connections.
- Support from family, friends, and the cultural community provided comfort and reduced loneliness and isolation.

**Potential impact:**

- Understanding how cultural dynamics intersect with the challenges of chronic kidney disease and kidney replacement therapy is essential for enhancing the delivery of culturally sensitive care, addressing health inequities, and improving quality of life.

## INTRODUCTION

Patients requiring kidney replacement therapy depend on lifelong treatment, which places major constraints on their ability to participate in social activities, and may negatively impact their social relationships. Poor social connections are associated with cardiovascular disease, hypertension, and inflammation, thereby increasing the risk of mortality in patients receiving kidney replacement therapy [1–4, 5]. Poor social connections may be experienced as social isolation (an objective low level of social contact) or loneliness (a subjective negative feeling where one desires greater social connections than they have) [6]. Studies have shown that >78% of patients receiving haemodialysis experience moderate to high levels of loneliness [7].

In high-income countries, minority groups including those from culturally and linguistically diverse (CALD) backgrounds face a higher burden of chronic kidney disease (CKD) and experience worse health outcomes [8]. It is also well established that CALD migrants and refugees commonly experience social isolation [9, 10], compounding the risk of poor health for those already vulnerable. However, evidence about the experience of social connections from the perspective of patients with CKD from CALD backgrounds is lacking. The Arabic-speaking population encompasses diverse cultural and linguistic groups originating from countries in the Middle East and North Africa. Arab patients may have distinct sociocultural norms and beliefs that shape their experiences of social connection and isolation [11]. Factors such as familial ties, religiosity, and community cohesion play pivotal roles in shaping social interactions and support networks [12]. Understanding how these cultural dynamics intersect with the challenges of CKD is essential for providing culturally sensitive care, enhancing disease management, improving quality of life, and understanding inequities in health outcomes. This study aims to describe the experiences and perspectives on social connections and social isolation among Arab patients with CKD receiving kidney replacement therapy, to inform the development of culturally tailored interventions and support services.

## MATERIALS AND METHODS

We followed the Consolidated Criteria for Reporting Qualitative Health Research framework [13].

### Participant selection and setting

We recruited participants from four large dialysis and transplant centres across the Western Renal Service (WRS), New South Wales, Australia. Patients with CKD from Arabic-speaking backgrounds receiving peritoneal dialysis (PD), haemodialysis (HD), or kidney transplant, aged ≥18 years, and could speak Arabic or English were eligible to participate. Potential participants were identified by renal nurses and clinic staff, and contacted by telephone. We used purposive sampling to capture a range of demographic and clinical characteristics (age, sex, type of treatment). Ethics approval was provided by the Western Sydney Local Health District Human Research Ethics Committee for the study (2021/ETH12248). All participants provided written informed consent.

### Data collection

The interview guide was developed based on a review of the literature and discussion among the research team, which included people with lived experience of kidney replacement therapy and people from Arabic backgrounds. The questions focused on topics including: experience of CKD, the impact of CKD on relationships and social connections, loneliness and social isolation, coping strategies, and suggestions (including interventions) to improve social connections (Supplementary File 1a and b). Author N.J., trained in qualitative methods and psychology, conducted a semi-structured interview with each participant by telephone between October 2023 and January 2024. Interviews were conducted in Arabic or English, depending on the participant's preference. Participant recruitment ceased when data saturation was reached. All interviews were audio-recorded, translated from Arabic and transcribed verbatim by N.J. who is bilingual in Arabic and English. Transcripts were offered to be shared with participants upon request; however, no participants requested their transcripts.

### Data analysis

The transcripts were analysed using thematic analysis [14]. Authors N.J and K.M. read and reviewed the interview transcripts, and N.J. completed line by line inductive coding in HyperRESEARCH software (version 4.5.6; ResearchWare Inc.) to identify concepts. Initial concepts were discussed among N.J. and K.M. and further classified into themes and subthemes. We used investigator triangulation whereby the preliminary themes and subthemes were discussed and revised among N.J., K.M., A.J., A.S., N.S.-R., and C.G.

## RESULTS

**Figure 1:**
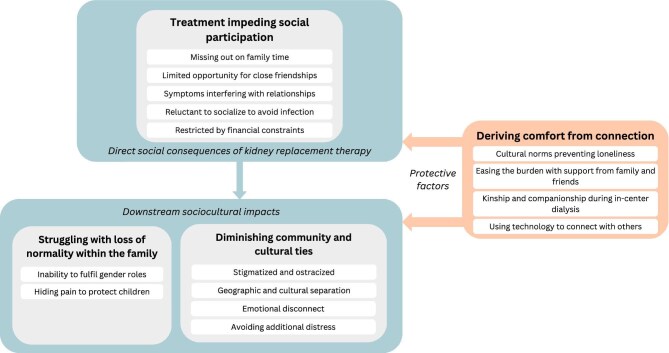
Thematic schema.

All 25 contacted patients participated in the study. Participant characteristics are provided in Table 1. Ten participants (40%) had received a kidney transplant, nine (36%) were receiving peritoneal dialysis, and six (24%) in-centre HD. Thirteen (52%) participants were male and 22 (88%) were born overseas. Five (20%) participants self-reported as having depression. Thirteen (52%) interviews were conducted in Arabic and 12 (48%) in English. The average duration of interviews was 22 minutes. We identified four themes: treatment impeding social participation, diminishing community and cultural ties, struggling with loss of normality within the family, and deriving comfort from connection. The respective subthemes are described below. Selected quotations illustrating each theme are provided in Table 2. A thematic schema depicting the relationship across themes is shown in Fig. 1.

**Table 1:** Participant characteristics (*n* = 25).

Characteristic	*n* (%)
Sex	
Male	13 (52)
Female	12 (48)
Age group (years)	
20–29	1 (4)
30–39	2 (8)
40–49	7 (28)
50–59	1 (4)
60–69	7 (28)
70–79	6 (24)
80–89	1 (4)
Treatment type	
Kidney transplant	10 (40)
Peritoneal dialysis	9 (36)
In-centre hemodialysis	6 (24)
Country of birth	
Lebanon	13 (52)
Australia	3 (12)
Egypt	3 (12)
Syria	3 (12)
Iraq	2 (8)
Sudan	1 (4)
Marital status	
Married/de facto	16 (64)
Partner (not living with)	0 (0)
Single	4 (16)
Divorced/separated	3 (12)
Widowed	2 (8)
Household status	
Living with family	24 (96)
Living alone	1 (4)
Employment status	
Full time	2 (8)
Part time/casual	3 (12)
Retired	11 (44)
Not employed	9 (36)
Highest level of education	
University	6 (24)
Diploma/TAFE	2 (8)
High school	7 (28)
Primary school	9 (36)
Other (none)	1 (4)
Comorbidities (self-report)	
Anaemia	4 (16)
Arthritis	1 (4)
Cancer	0 (0)
Depression	5 (20)
Diabetes	10 (40)
Heart disease	7 (28)
Hypertension	14 (56)
Thyroid disease	5 (20)

**Table 2:** Selected quotations to support each theme.

Theme	Selected quotations
Treatment impeding social participation	
Missing out on family time	‘It's the family. One time they went to Queensland so I didn't go because of my dialysis [at the time]. I felt a little bit sad … because I really wanted to go with them, but because of my dialysis, I couldn't leave’. (Transplant, female aged 25) ‘You don't go anywhere. You don't see your siblings. You don't see your family … You feel lonely’. (HD, female aged 64) ‘When my children go out for a holiday, I can't go with them. When someone [family member] goes to the beach for a day, and they ask me to come, I can't do it. I can't go in the water’. (PD, male aged 67)
Limited opportunity for close friendships	‘[When I was on dialysis] I had to always be at home at certain times to do that dialysis. I couldn't go camping or weekends and all that stuff. I couldn't just go with my friends’. (Transplant, male aged 42) ‘I'm just sitting at home most of the time and they [my friends] understand, but of course, because I'm not with them that much I lost being close with them’. (Transplant, male aged 42) ‘The only thing is sometimes say my friends want to do something on a particular day and I have to do dialysis. So I have to arrange my plan around my dialysis, which is annoying’. (Transplant, male aged 40)
Symptoms interfering with relationships	‘I know that kidney disease makes it very, very hard to start new relationships. Very, very, hard. whether it's a friend or a lover or whatever it is, it makes it very difficult … they are expecting you to do this and to do that while I did not have the strength to do this … So you know it does get in the way of making new relationships, very severely, very severely with a lover. Because you think oh they … Like I can't work at the moment. I can't do this. I can't do that. Inferior. So yes. It makes it very hard to start new relationships’. (HD, male aged 46) ‘It is really tough for the relationships because you're always tired. You're always unable to do a lot of things … your partner will be impacted a lot because there's a lot of things when they're younger, they wanted to do and they might not be able to do’. (Transplant, male aged 42)
Reluctant to socialize to avoid infection	‘I can't really fix the travel part, but try to travel as minimal as I can, but did travel with the help. It was the help of the company that supported the dialysis stuff. So, I was able to travel a couple of times making sure that the area that we are going to is sterilized because of the infection and stuff. So yeah, I tried but it wasn't easy’. (Transplant, male aged 42) ‘I was pretty much in isolation from the start [of COVID-19] because avoid people coming over and seeing me and all that stuff. So for two years I didn't see anyone except my mum, dad and sister … I got out of isolation and the lockdown with a lot of anxiety’. (Transplant, female aged 30)
Restricted by financial constraints	‘I spend ${\$}$800 on medicine and bills. And by the time day 10 comes the 800’s gone and [then for] four five days I've got nothing in my pocket. If I wanted to walk up the road to buy a hamburger and chips, guess what, I've got no money left’. (PD, male aged 67) ‘I could start doing things when I know my bills are paid. And I don't have to worry about it. Because that's what worries me the most, you know, can't do this, can't do that. Haven't got enough money. I’ve got to pay this bill. I’ve got to pay that bill’. (PD, male aged 60)
Diminishing community and cultural ties	
Stigmatized and ostracized	‘They [relatives] stopped calling me. They started to make up stories about how busy they are’. (Transplant, female aged 42) ‘[Social isolation] is inevitable. They [friends] used to come to me to enjoy. Now I need someone to look after me. What can I do? I sit alone, me and this man [husband]’. (PD, female aged 62) ‘My brain is always thinking what was the wrong that I did? Why have the people rejected me? My brain is always busy with thoughts’. (PD, female aged 46) ‘When they [other people] knew [about CKD] they sort of drifted away’. (PD, male aged 67)
Geographic and cultural separation	‘I [would] love to go to Lebanon. I would like if there was support for me to know how to deal with chronic kidney disease and they book for me everything. I want to see and remember my life there, my friends and cousins’. (PD, male aged 79) ‘I’ve been treating my kidney for almost 15 years. It's been 15 years that I have not gone anywhere. I am stuck at home. I cannot go anywhere. I cannot see my siblings overseas. I cannot see my family. I’m sitting at home’. (HD, female aged 64) ‘My family went on these holidays and all I could do was just look at the WhatsApp and the pictures they were sending. I cried. I sat crying because I wanted to be there. My family was going overseas to visit family and I was just watching the photos they were sending and again crying’. (HD, male aged 46) ‘I feel lonely because I am Sudanese. My kids grew up here. They don't know how to make Sudanese food and I’m tired. Sometimes I crave Sudanese food but I can't make it and my kids don't know how to make it’. (PD, female aged 46)
Emotional disconnect	‘I still could be sitting around my group of friends and still feel lonely because I’m going through something that again they wouldn't understand’. (Transplant, female aged 30) ‘You feel so alone because no matter how much support someone gives you, no matter how much love they show you, they are not going through what you're going through’. (HD, male aged 46) ‘Being sick and having to do dialysis, people don't understand that. They don't understand what dialysis is, fully’. (PD, male aged 67)
Avoiding additional distress	‘Well, actually what you suggested is good talking to other patients, but I don't know how good it would be to talk to other patients who are terribly sick, because especially someone is new, that would scare the hell out of me. Maybe talk to, I don't want to brag, talk to someone like me. Talk to someone who's doing well’. (HD, male aged 46) ‘I know a relative with CKD who was so depressed and he used to say I will not live, I will not live. But then he did the transplant and is living a good life. When I used to see them depressed, I didn't like to interact with them … They are not in the mood of talking’. (Transplant, male aged 48) ‘I have already got a problem and I don't want to hear other people's problems. I've got a lot of problems on my mind to hear other people's problems’. (PD, male aged 60)
Struggling with loss of normality within the family	
Inability to fulfil gender roles	‘When they attached the tubes to my belly [for the PD], I wasn't allowing him [husband] to see me. And you know, he is a man, he wants his wife, but I kept rejecting, rejecting, rejecting. That was a very difficult time for my mental health’. (Transplant, female aged 42) ‘It's been two years that I cannot shower. I cannot walk to the shower … I need to shower every day. It's been two years my wife is showering me. She is showering me. Currently, I'm 73 years old. From this age, my wife is showering me? What kind of life is this? This is not a life … I was working in the countryside, strong and happy. I was able to feed my family just like other people. Now I cannot feed my family like other people’. (PD, male aged 73)
Hiding pain to protect children	‘They [my daughters] always ask me, how are you? How have you been? I can't take that off my face, if you know what I mean. I am always in a terrible state.’(PD, male aged 75) ‘I have been in really tough times, but I was telling my daughter that I am alright. I’m good. Don't worry about me’. (Transplant, female aged 42) ‘I also sacrificed for my only son. I thought I should not be depressed, for my son’. (Transplant, male aged 48)
Deriving comfort from connection	
Cultural norms preventing loneliness	‘Being in the Egyptian community you don't really get loneliness or the word itself. I know the definition of it is like being isolated. That's fine, but I’m always with my family, my wife and my friends. If I can't, they always give me a call or I call them or see them at church or something like that. I don't put myself into that category at all’. (Transplant, male aged 42) ‘They tell me, but I don't feel it [loneliness] because I have a home and am happy with my children. But I sometimes see other women complaining to me that they are living alone and don't have anyone. They have children, but they don't visit them as they are busy with their jobs’. (HD, female aged 66)
Easing the burden with support from family and friends	‘Here [in Australia] there is my daughter and her in laws, and the extended family of her husband. We have people here, we have relatives. There are people who we know. When we first arrived [to Australia], all of them invited us and celebrated our arrival. We enjoyed at the beginning, we enjoyed a lot. Although I was hospitalized since the first week of our arrival. I celebrated Eid it at the hospital. It was the big festive. I spent it at the hospital but I was happy because everyone was around me’. ‘You are in the room dialysing, but yeah, I got company. My dad, you know, spends time with me. We watch TV. It's not so bad’. (Transplant, male aged 40) ‘My older sister did not leave me. All my siblings were good. My sister used to care for me and pick me up [from dialysis]. My wife became my carer and was looking after me’. (Transplant, male aged 48)
Kinship and companionship during in-centre dialysis	‘There are women with me in the centre who are originally from the same town in Lebanon … When I first started the dialysis, I didn't know anyone, but they told me there was a woman who I knew was there. When I talked with her, I realized that she knew the other women. I joined them and started talking to them … All of them are from my town … we enjoy ourselves. This is better … We’d talk about whatever comes to our minds. We spend the time’. (HD, female aged 70). ‘We [she and other Lebanese patients] talk and eat sunflower seeds … It is nice. They must have missed me for this week’. (HD, female aged 75)
Using technology to connect with others	‘Social media these days, it's very easy to get them to talk to you or you talk to them. Even if you're not next to them. So yeah, we have COVID for three years and I never felt lonely, I guess at that time as well. So even when we socially distant’. (Transplant, male 42) ‘I posted [on Facebook patient group] when I got my transplant and when I had all my complications and you know everyone's very, very supportive and they you know they try to help you through it and like give you advice of the same thing that they’ve been through, but a lot of people message as well’. (Transplant, female aged 30) ‘It [my phone] is always next to me. My daughter rings me the most. She calls me twice daily). (HD, female aged 64)

### Treatment impeding social participation

#### Missing out on family time

Participants receiving dialysis reported missing out on activities and time with family as they were ‘attached to a machine and you can't really move around or do anything’ (HD). In particular, parents receiving dialysis were sad and distressed from being unable to spend enough time with their children. One mother reported changing her own dialysis schedule so that she could accompany her child during the day—‘when my daughter had something during the day, I was waking up during the night to do the dialysis every three hours at night so I did not need the machine [during the day]’.

#### Limited opportunity for close friendships

Participants receiving dialysis reported how being unable to travel and attend social gatherings caused them to ‘lose relationships’. This was particularly important for younger patients who lost opportunities to develop close bonds with their friends—‘not so much as losing friends, but not having close relationship with friends. Gradually, after 10 years [of dialysis], I lost being close to my friends because I don't go out anymore’. After transplant, the social life of participants had ‘kind of gone back to normal’ but that ‘what you missed at that time [of youth], that social life is already missed … you're not going to get that back’.

#### Symptoms interfering with relationships

Kidney disease made it ‘very, very hard to start new relationships, whether it's a friend or a lover’. Fatigue would ‘get in the way of making new relationships’ as they felt tired and unable to meet others’ expectations—‘you always feel like you’re dying tired and someone who's new [to the relationship] is not used to that’. Fatigue, nausea and dizziness among patients receiving dialysis prevented them from attending social activities: ‘if they [friends] wanted to go on a hike that I knew requires energy that I didn't have, I feel like I’ve missed out on some of those things because I didn't have the energy to do it’.

#### Reluctant to socialize to avoid infection

Kidney transplant recipients were afraid of getting infections when leaving the house, travelling and socializing. A participant who was receiving HD during the COVID-19 pandemic was ‘very afraid’ of socializing for fear of contracting infection, which significantly impaired her mental health. They ‘avoided people coming over and seeing me and all that stuff. For two years, I didn't really see anyone except my mum, dad and sister … I get out of isolation, with the lockdown and stuff with a lot of anxiety’.

#### Restricted by financial constraints

Out-of-pocket treatment costs for certain medications, along with general household expenses and bills, limited some participants’ ability to engage in activities. One participant explained: ‘I could start doing things when I know my bills are paid. And I don't have to worry about it. Because that's what worries me the most, you can't do this, can't do that’. Another participant highlighted the inadequacy of government financial assistance, noting that it often did not last until the next payment cycle: ‘I spend ${\$}$800 on medicine and bills. And by the time day 10 comes the 800’s gone and [then for] four five days I've got nothing in my pocket’.

### Diminishing community and cultural ties

#### Stigmatized and ostracized

Participants felt excluded from their community after their CKD diagnosis became known, stating that others stopped contacting them and ‘made up stories about how busy they are’. As such, patients felt distressed and rejected—‘What was the wrong that I did? Why have the people rejected me?’ One transplant recipient stated that her community members avoided interacting with her as they did not want to develop CKD—‘Being Lebanese, OK, you can understand them. They say I don't want that [CKD] to happen to me’.

#### Geographic and cultural separation

Some participants felt socially isolated living in Australia, away from their families overseas. Participants were lonely and sad when they could not accompany their immediate family on overseas trips to visit their extended family due to dialysis or if they were awaiting a kidney transplant. The only thing they could do was to ‘watch the photos they [family] were sending and crying’ (HD). Participants also emphasized the importance of food as a connection to their culture and link to their home country, and expressed a sense of disconnection when they could not cook traditional foods due to symptoms such as fatigue—‘My kids grew up here. They don't know how to make Sudanese food and I’m tired … if my friends were here I would have asked them, [but] there's no help and there is no energy. There's nothing’.

#### Emotional disconnect

Participants who were receiving dialysis or had a transplant felt they were the only person in their social network with CKD. Feelings of loneliness endured even when participants were well supported or among family and friends, as others could not understand or relate to their experience—‘You feel so alone because no matter how much support someone gives you, no matter how much love they show you, they are not going through what you’re going through’ (HD).

#### Avoiding additional distress

Some participants wanted to distance themselves from other patients with CKD who were not physically or mentally well, as they were already overwhelmed by their own health challenges and wanted to protect themselves from additional stress. Some patients felt that if they were new to CKD or dialysis and spoke with someone ‘terribly sick … that would scare the hell out of me’. Other patients felt that they ‘already got a problem and don't want to hear other people's problems’ (PD).

### Struggling with loss of normality within the family

#### Inability to fulfil gender roles

Participants mentioned that CKD disrupted traditional gender roles in the family and impaired intimate relationships between spouses. Some men felt shame in no longer being able to provide for their family—‘I hate myself because I cannot do anything’. Women felt embarrassed and overwhelmed by the appearance of the catheters and tubing that lowered their confidence in relationships, which led to preventing their husbands from seeing their bodies—‘he is a man, he wants his wife, but I kept rejecting’ (PD).

#### Hiding pain to protect children

Parents, when interacting with their children, tried to conceal feelings of pain and discomfort to prevent their children from worrying, which in turn led to a reduced closeness in relationships. A kidney transplant recipient stated: ‘I have been in really tough times, but I was telling my daughter that I am alright. I’m good. Don't worry about me’.

### Deriving comfort from connection

#### Cultural norms preventing loneliness

Being part of a cultural community helped participants to feel supported and prevented loneliness or social isolation. One transplant recipient mentioned that ‘being in an Egyptian community you don't really get loneliness or the word itself’, explaining how the term ‘loneliness’ does not exist in Arabic as it does in English. Similarly, a Lebanese kidney transplant recipient highlighted how being socially connected was a cultural norm within the Lebanese community, stating ‘you should know with the Lebanese, everyone is around them’. Some patients described the definition of loneliness ‘like being isolated’ and noted that patients without sufficient support may experience this—‘I sometimes see other women complaining to me that they are living alone and don't have anyone. They have children, but [the children] don't visit them’.

#### Easing the burden with support from family and friends

Support from friends and family eased the burden of CKD and reduced loneliness—‘Even when I was there learning the [dialysis] machine, I used to have friends come past the hospital and sit with me’. Some emphasized the importance of support from family over that of friends—‘A good friend might help. However, I do not think it can help as the family does … Your family, your children, aunties, uncles. Those are the important ones’. Further, participants underscored the importance of children and immediate family members over that of the extended family: ‘If someone is excluded by their family and they are not sought after or concerned about, particularly by their children, this can lead to loneliness. The neglect from aunts, uncles, and other distant relatives is not as impactful as when one's children and close relatives do not show care’.

#### Kinship and companionship during in-centre dialysis

Developing relationships and connections with other patients during in-centre HD sessions, who shared the same language and cultural background, provided comfort and companionship to the participants. Some patients discovered that there were others at their centre ‘who were originally from the same town’, which created strong bonds and kinship: ‘I joined [the women] and started talking to them … All of them are from my town … We enjoy ourselves’. Another female patient explained that during the in-centre dialysis sessions, she and the other patients would ‘talk and eat sunflower seeds … it is nice’ (HD).

#### Using technology to connect with others

Participants used their mobile phones and social media as coping strategies for loneliness and isolation by connecting with friends and relatives. One participant emphasized the importance of the mobile phone in connecting with her children when they couldn't physically be present, due to treatment or issues with mobility—‘it [my mobile] is always next to me. My daughter rings me the most. She calls me twice daily’ (HD). One kidney transplant recipient utilized social media to remain socially connected with his friends, stating: ‘Social media these days, it's very easy to get them [friends] to talk to you or you talk to them’.

## DISCUSSION

Patients with CKD from Arab communities faced a myriad of barriers to social participation due to their kidney replacement therapy and associated symptoms. The reduced social participation and the physical and emotional impacts of kidney replacement therapy led to downstream impacts on participants’ sociocultural connections. Within families, the disruption to social and gender roles caused anguish among spouses and parents who wanted to protect their children from the emotional burden of CKD. Among communities and cultural groups, participants lost a sense of social belonging and felt isolated as they experienced stigma around their CKD, were unable to travel to their home countries due to their kidney replacement therapy, and were impaired by symptoms including fatigue which limited their ability to connect with their culture through activities such as cooking. While public hospital health care (including dialysis) is generally free in Australia, some participants noted that out-of-pocket treatment costs for certain medications and other expenses limited their ability to engage in social activities. Participants described how support from family, friends, and their broader cultural community helped to provide them comfort and mitigate feelings of loneliness or isolation.

There were notable differences in the experiences of loneliness and social isolation among patients with CKD across treatment modalities. Patients receiving dialysis, whether home-based PD or in-centre HD, described missing out on shared activities with family and friends, including social events and travel, which exacerbated feelings of loneliness. Limitation of travel was a particularly important factor influencing social isolation for this population given that the majority of participants were migrants with close family and friends overseas. Unlike for patients receiving home-based dialysis, Arabic-speaking patients receiving in-centre dialysis had opportunities during dialysis sessions to encounter other patients from similar cultural or language backgrounds, which helped patients develop a sense of connection and reduced feelings of loneliness. For kidney transplant recipients, some patients noted improvements in energy levels and increased opportunities for social participation after transplantation. However, other kidney transplant recipients emphasized that receiving a transplant in adulthood could not make up for the lost opportunities to build strong social connections during their youth. Participants explained that youth is a critical period for developing long-term friendships, and their CKD and kidney replacement therapy, especially during childhood and early adulthood, hindered this process.

Our study reveals findings that relate to the broader CKD population, yet it also uncovers aspects unique to the Arab population with CKD. Traditionally, Arab values such as privacy and modesty can influence the extent to which members of these communities communicate their personal or health needs [15]. In this study, we found themes relating to a lack of honesty, vulnerability, and confidence in role performance and identity within marriages and families, which may act as barriers to intimacy and emotional connection. Studies have also highlighted that among Arab societies, as with many Asian, African, and Latin cultures, family and community are central aspects of the well-being of this population [16]. In this study, we found that some participants felt shunned from their communities which caused them major distress, while others were more cognizant of the cultural stigma surrounding their disease and could understand the reasons for their social exclusion. A lack of awareness and knowledge of CKD within Arab communities has been reported in several studies, and there is a need to increase CKD literacy in this community, particularly to help reduce the stigma associated with the disease [17,18].

Food and traditional diets emerged as important connections to culture in this study. A systematic review of qualitative studies investigating treatment burden for patients with CKD found that among immigrant communities, including Mexican Americans and South Asians in Europe, preserving traditional food and cooking customs was an important way to prevent being stigmatized by others in their community [19]. Patients reported that dietary restrictions could be mistaken for poverty, leading to judgement from their community [20].

This study has several implications for practice, including implementing cultural peer support, family counselling and enhancing community awareness. Offering opportunities for patients to engage with others from similar cultural backgrounds could aid in preventing or alleviating loneliness and social isolation. Implementing one-on-one peer mentoring or group support initiatives within clinics has been shown to improve quality of life, psychological well-being, and treatment adherence for patients [21–23], particularly in cases where both patients and mentors share Arabic heritage, as the Arab culture values a collaborative approach to health-related decision-making [24]. Depending on the patient's values and preferences, utilizing culturally tailored family counselling may help to address relational challenges among patients, spouses and children [25]. Since participants in this study highlighted the benefits of technology and social media for staying connected, creating online platforms or forums where individuals can share experiences and access resources should be explored. These platforms could be especially valuable for people with mobility issues or those living in remote areas. Finally, integrating assessments of loneliness and social isolation into routine clinical care could enable early identification of psychosocial issues and help prevent negative health outcomes [26].

Although participants in this study did not explicitly identify barriers to accessing health or social services, it is well established that refugee and minority communities face multiple barriers to care, including delays in referral processes and administrative issues [27]. For example, patients from countries with limited healthcare infrastructure (e.g. Syria, Iraq) may struggle to obtain essential medical documents or may be unaware of the available options for kidney replacement therapy [28]. Healthcare providers should ensure that additional supports are provided to these patients to help address language barriers and access to relevant services and programmes [29].

This study is grounded in the lived experiences of Arab patients with CKD receiving kidney replacement therapy in Australia; however, there are some potential limitations. One limitation of this study is the small sample size, which may not fully capture the entire range of experiences within this population. All interviews were conducted by telephone, which may have limited the level of rapport established between the interviewer and the patients. As most patients were located at home during the interview, it is unknown whether the presence of other household members may have influenced the information disclosed.

Patients with CKD from Arab backgrounds encounter substantial challenges to social participation stemming from their kidney replacement therapy. Patients experience emotional strain and disruption of family dynamics, particularly among spouses and parents. Patients felt isolated by the stigma surrounding CKD and were hindered by fatigue, which limits travel and engagement in cultural activities. Nonetheless, support from family, friends, and the larger community provided comfort. There is a need for increased initiatives in clinical practice and policy to tackle poor social connections, fostering improved patient-centred care and outcomes among culturally and linguistically diverse patients with CKD.

## Supplementary Material

sfaf081_Supplemental_File

## Data Availability

The data that support the findings of this study are available from the corresponding author upon reasonable request.

## References

[1] Holt-Lunstad J, Smith TB, Baker M et al. Loneliness and social isolation as risk factors for mortality: a meta-analytic review. Perspect Psychol Sci 2015;10:227–37. 10.1177/1745691614568352 25910392

[2] Hawkley LC, Thisted RA, Masi CM et al. Loneliness predicts increased blood pressure: 5-year cross-lagged analyses in middle-aged and older adults. Psychol Aging 2010;25:132. 10.1037/a0017805 20230134 PMC2841310

[3] Brown EG, Gallagher S, Creaven AM. Loneliness and acute stress reactivity: a systematic review of psychophysiological studies. Psychophysiology 2018;55:e13031. 10.1111/psyp.13031 29152761

[4] Valtorta NK, Kanaan M, Gilbody S et al. Loneliness and social isolation as risk factors for coronary heart disease and stroke: systematic review and meta-analysis of longitudinal observational studies. Heart 2016;102:1009–16. 10.1136/heartjnl-2015-308790 27091846 PMC4941172

[5] Iseki K, Shinzato T, Nagura Y et al. Factors influencing long-term survival in patients on chronic dialysis. J Clin Exp Nephrol 2004;8:89–97. 10.1007/s10157-004-0285-z 15235924

[6] Holt-Lunstad J . The major health implications of social connection. Curr Dir Psychol Sci 2021;30:251–9. 10.1177/0963721421999630

[7] Pallone JM, dos Santos DGM, Oliveira Dias AL et al. Loneliness level and its associated factors in patients with hemodialysis. Clin Nurs Res 2022;31:1164–71. 10.1177/10547738211061447 34955033

[8] Garcia-Garcia G, Jha V. CKD in disadvantaged populations. Kidney Int 2015;87:251–3. 10.1038/ki.2014.369 25635713

[9] Atwell R, Correa-Velez I, Gifford S. Ageing out of place: health and well-being needs and access to home and aged care services for recently arrived older refugees in Melbourne, Australia. Int J Migration Health Soc Care 2007;3:4–14. 10.1108/17479894200700002

[10] Georgeou N, Schismenos S, Wali N et al. A scoping review of aging experiences among culturally and linguistically diverse people in Australia: toward better aging policy and cultural well-being for migrant and refugee adults. Gerontologist 2023;63:182–99. 10.1093/geront/gnab191 34969076 PMC9872767

[11] Dew A, Lenette et al. ‘To the Arabic community disability is not normal’: multiple stakeholder perceptions of the understandings of disability among Iraqi and Syrian people from refugee backgrounds. J Refugee Studies 2021;34:2849–70. 10.1093/jrs/feaa111

[12] Khaled SM, Amro I, Bader L et al. Factors associated with depression and anxiety in the adult population of Qatar after the first COVID-19 wave: a cross-sectional study. Discover Psychol 2021;1:9. 10.1007/s44202-021-00009-z PMC8686347 40477870

[13] Tong A, Sainsbury P, Craig J. Consolidated criteria for reporting qualitative research (COREQ): a 32-item checklist for interviews and focus groups. Int J Qual Health Care 2007;19:349–57. 10.1093/intqhc/mzm042 17872937

[14] Braun V, Clarke V. Using thematic analysis in psychology. Qual Res Psychol 2006;3:77–101. 10.1191/1478088706qp063oa

[15] Al-Naimi L, Alistar M. Understanding cultural and religious values relating to awareness of women's intimate health among Arab Muslims. Proceedings of the 2024 CHI Conference on Human Factors in Computing Systems. Honolulu: Association for Computing Machinery, 2024, Article 690.

[16] Allam I, Gresham M, Phillipson L et al. Beliefs around help-seeking and support for dementia in the Australian Arabic speaking community. Dementia 2023;22:995–1009. 10.1177/14713012231166170 36990452 PMC10262330

[17] Khalil A, Abdalrahim M. Knowledge, attitudes, and practices towards prevention and early detection of chronic kidney disease. Int Nurs Rev 2014;61:237–45. 10.1111/inr.12085 24571391

[18] Alobaidi S . Knowledge of chronic kidney disease among the population of Saudi Arabia evaluated using a validated questionnaire: a cross-sectional study. Patient Prefer Adherence 2021;15:1281–8. 10.2147/PPA.S315369 34163145 PMC8214335

[19] Roberti J, Cummings A, Myall M et al. Work of being an adult patient with chronic kidney disease: a systematic review of qualitative studies. BMJ Open 2018;8:e23507. 10.1136/bmjopen-2018-023507 PMC6129107 30181188

[20] de Brito-Ashurst I, Perry L, Sanders TAB et al. Barriers and facilitators of dietary sodium restriction amongst Bangladeshi chronic kidney disease patients. J Hum Nutr Diet 2011;24:86–95. 10.1111/j.1365-277X.2010.01129.x 21114553

[21] Ghahramani N, Chinchilli VM, Kraschnewski JL et al. Effect of peer mentoring on quality of life among CKD patients: randomized controlled trial. Kidney Dis 2021;7:323–34. 10.1159/000514477 PMC8314752 34395547

[22] Longley RM, Harnedy LE, Ghanime PM et al. Peer support interventions in patients with kidney failure: a systematic review. J Psychosom Res 2023;171:111379. 10.1016/j.jpsychores.2023.111379 37270909 PMC10340538

[23] Cervantes L, Rizzolo K, Carr AL et al. Social and cultural challenges in caring for Latinx individuals with kidney failure in urban settings. JAMA Netw Open 2021;4:e2125838. 10.1001/jamanetworkopen.2021.25838 34533567 PMC8449281

[24] Alzubaidi H, McNamara K, Browning C et al. Barriers and enablers to healthcare access and use among Arabic-speaking and Caucasian English-speaking patients with type 2 diabetes mellitus: a qualitative comparative study. BMJ Open 2015;5:e008687. 10.1136/bmjopen-2015-008687 PMC4654379 26576809

[25] Berry E, Davies M, Dempster M. Exploring the effectiveness of couples interventions for adults living with a chronic physical illness: a systematic review. Patient Educ Couns 2017;100:1287–303. 10.1016/j.pec.2017.02.015 28228340

[26] Fildes K, Stefoska-Needham A, Atkinson J et al. Optimising health care for people living with chronic kidney disease: health-professional perspectives. J Ren Care 2022;48:168–76. 10.1111/jorc.12409 35094501

[27] Norton JM, Moxey-Mims MM, Eggers PW et al. Social determinants of racial disparities in CKD. J Am Soc Nephrol 2016;27:2576–95. 10.1681/ASN.2016010027 27178804 PMC5004663

[28] Lemke J, Schild R, Konrad M et al. Distribution and management of the pediatric refugee population with renal replacement: a German pediatric cohort. Pediatr Nephrol 2021;36:271–7. 10.1007/s00467-019-04374-9 31897711

[29] Gregory E, Woo TKW, Hategan A. Cultural considerations when caring for racial and ethnic minority patients with end-stage renal disease. In: Hategan A, Bourgeois JA, Gangji AS, Woo TKW (eds). Psychonephrology: A Guide to Principles and Practice. Cham: Springer, 2022, 377–94.

