# Supplementary material for: Perspectives on social health among patients from Arab backgrounds receiving kidney replacement therapy: an interview study
Source: Clin Kidney J. 2025 Mar 13;18(4):sfaf081. doi: 10.1093/ckj/sfaf081 (PMC11982812; doi:10.1093/ckj/sfaf081)
Supplement: sfaf081_Supplemental_File [file sfaf081_supplemental_file.docx]

**Supplementary File 1a. Interview Guide – English**

**1. Experience of chronic kidney disease**

- Could you tell me about how and when you first found out you had kidney disease?

**2. Impact on relationships and social life**

- Thinking of your family, friends, or broader social networks, what were the biggest or main impacts of having CKD on your relationships or social life? Has this changed over time?
- Did you expect any changes to your relationships with others when you were first diagnosed? How did you think/imagine your relationship with them would change?
- Have you established new relationships (friendships, other patients) – how has this impacted you?

**3. Loneliness or isolation**

- How would you define social isolation? What does the term “social isolation” mean to you?
- What about loneliness? What does the word “loneliness” mean to you?
- How important do you think is the issue of loneliness and isolation in patients with kidney disease – what are the reasons for your view on this?
- What do you think may contribute to patients with CKD feeling lonely or isolated? - what might make it worse?
- What do you think are the impacts of loneliness or isolation in patients with kidney disease? *(e.g. outlook, how they manage their health, effect of lifestyle, ability to establish new relationships)*
- Have you felt lonely or socially isolated at times as a result of your CKD – could you give me an example? Was there anything you did to help overcome this, if so what did you do?

**4. Coping strategies and preferred interventions**

- What do you think about services that connect you with other patients with CKD, is this something that would be of interest to you? Why/why not?
- What kind of things would you normally do (or avoid doing) to manage your feelings of loneliness or isolation? What other activities/services would help with your loneliness?
- What kind of services do you think would help other patients with chronic kidney disease (e.g. patient support groups, counselling, online discussion forums, referral to community recreation activities)?
- Is there something you would like to add that you think might be relevant?

**Close**

**Supplementary File 1b. Interview Guide - Arabic**

**دليل المقابلة**

**تجربة مرض قصور الكلى المزمن.1**

- هل يمكنك أن تخبرني عن كيفية اكتشافك لمرض الكلى ومتى اكتشفته لأول مرة؟

**التأثير على العلاقات والحياة الاجتماعية. 2**

- بالنسبة لعائلتك وأصدقائك وشبكاتك الاجتماعية الأوسع، ما هي أكبر التأثيرات أو التأثيرات الرئيسية لمرض *قصور* الكلى المزمن على علاقاتك أو حياتك الاجتماعية؟ هل تغير ذلك مع مرور الوقت؟
- هل كنت تتوقع أي تغييرات في علاقتك مع الآخرين عندما تم تشخيصك للمرة الأولى؟ كيف كنت تعتقد / تتخيل أن علاقتك بهم ستتغير؟
- هل كونت علاقات جديدة (صداقات، مرضى آخرين)؟ كيف أثر ذلك عليك؟

**3. الوحدة أو العزلة**

- كيف تعرّف العزلة الاجتماعية؟ ماذا يعني لك مصطلح "العزلة الاجتماعية"؟
- وماذا عن الوحدة؟ ماذا يعني لك كلمة "الوحدة"؟
- كم هي مهمة برأيك قضية الوحدة والعزلة في حالة مرضى الكلى - ما هي الأسباب التي تجعلك تؤيد هذا الرأي؟
- ماذا الذي يساهم في شعور مرضى قصور الكلى بالوحدة أو العزلة؟ وما الذي قد يزيد من حدة هذا الشعور؟
- برأيك ما هي تأثيرات الوحدة أو العزلة على مرضى قصور الكلى؟ (مثلاً، كيف يؤثر على توجههم، وكيف يديرون صحتهم، وتأثيره غلى أسلوب حياتهم، وقدرتهم على إقامة علاقات جديدة)
- هل شعرت يومًا بالوحدة أو العزلة الاجتماعية بسبب مرض قصور الكلى؟ هل يمكنك أن تعطينى مثالًا؟ هل كنت تقوم بأي شيء للتغلب على ذلك؟ إذا كان الأمر كذلك، فماذا فعلت؟

**4. استراتيجيات التأقلم والتدخلات المفضلة**

- ما رأيك في الخدمات التي تربطك بمرضى قصور الكلى المزمن الاخرين؟ هل هذا شيء يثير اهتمامك؟ لماذا / لماذا لا؟
- ما هي الأشياء التي تقوم بها عادة (أو تتجنب القيام بها) للتعامل مع مشاعر الوحدة أو العزلة؟ ما هي الأنشطة / الخدمات الأخرى التي قد تساعدك في التغلب على الوحدة؟
- ما هي الخدمات التي تعتقد أنها ستساعد مرضى قصور الكلى المزمن الآخرين؟ (مثلا مجموعات دعم المرضى، والاستشارة النفسية، ومنتديات النقاش عبر الإنترنت، والإحالة إلى أنشطة ترفيهية في المجتمع)
- هل هناك شيء ترغب في إضافته وتعتقد أنه قد يكون ذا صلة؟

**ختام**
